# Evaluation of cancer-derived myocardial impairments using a mouse model

**DOI:** 10.18632/oncotarget.27759

**Published:** 2020-10-13

**Authors:** Yoshihiro Miyagawa, Shota Nukaga, Takuya Mori, Rina Fujiwara-Tani, Kiyomu Fujii, Shiori Mori, Kei Goto, Shingo Kishi, Takamitsu Sasaki, Chie Nakashima, Hitoshi Ohmori, Isao Kawahara, Yi Luo, Hiroki Kuniyasu

**Affiliations:** ^1^Department of Molecular Pathology, Nara Medical University, Kashihara, Nara 634-8521, Japan; ^2^Division of Rehabilitation, Hanna Central Hospital, Ikoma, Nara 630-0243, Japan; ^3^Division of Rehabilitation, Hoshida Minami Hospital, Katano, Osaka 576-0022, Japan; ^4^Key Laboratory of Neuroregeneration of Jiangsu and Ministry of Education, Co-Innovation Center of Neuroregeneration, Nantong University, Nantong, Jiangsu Province 226001, China

**Keywords:** cachexia, myocardium, atrophy, mitochondria, oxidative stress

## Abstract

Myocardial damage in cancer patients is emphasized as a cause of death; however, there are not many murine cachexia models to evaluate cancer-derived heart disorder. Using the mouse cachexia model that we established previously, we investigated myocardial damage in tumor-bearing mice. In cachexic mice, decreased heart weight and myocardial volume, and dilated left ventricular lumen, and atrophied cardiomyocytes were noted. The cardiomyocytes also showed accumulated 8-hydroxydeoxyguanosine, decreased leucine zipper and EF-hand-containing transmembrane protein-1, and increased microtubule-associated protein light chain3-II. Levels of tumor necrosis factor-α and high-mobility group box-1 proteins in the myocardium were increased, and nuclear factor κB, a signaling molecule associated with these proteins, was activated. When rat cardiomyoblasts (H9c2 cells) were treated with mouse cachexia model ascites and subjected to flux analysis, both oxidative phosphorylation and glycolysis were suppressed, and the cells were in a quiescent state. These results are in good agreement with those previously reported on cancerous myocardial damage. The established mouse cachexia model can therefore be considered useful for analyzing cancer-derived myocardial damage.

## INTRODUCTION

Cachexia affects 40–80% of all patients with advanced cancer, especially those with pancreatic, gastric, and esophageal cancers [1–3]. Moreover, cachexia accounts for 20–30% of all cancer-related deaths [4]. Although weight loss is an important phenotype of cachexia, weight loss in cancer patients is associated with myocardial atrophy [5, 6]. Therefore, it is considered that myocardial atrophy is one of the phenotypes of cancer cachexia. In fact, myocardial damage is a common cause of cancer death [7].

Cancer-derived myocardial impairment is a status wherein cardiac atrophy, remodeling, and dysfunction are integrated [8, 9]. Cancer-derived myocardial impairment is characterized by morphological alterations such as left ventricular (LV) wall thinning, decreased heart volume, myocardial fibrosis, and remodeling of the left ventricle as reported in gastrointestinal, pancreatic, and non-small cell lung cancer [8]. The causes of cancer-derived myocardial impairment might be the effects of cancer itself, background heart disease, and influence of cancer treatments; however, they have not been given much clinical importance, and specific treatment efforts are delayed [8].

Various factors have been reported as the causes of cancer-derived myocardial impairment derived from the cancer itself. These factors are cancer-induced cytokines, oxidative stress, depletion of antioxidants, and protein catabolism due to AKT/mTOR inhibition [10]. Moreover, energy metabolism disorder due to mitochondrial dysfunction is considered one of the causes of cancer-derived myocardial impairment [8]. Mitochondrial dysfunction in cancer has been reported to occur in the form of mitochondrial uncoupling, reduced ATP production, and NFkB-MAPK-dependent mitochondrial disorder [11, 12]. In addition, it has been reported that cardiomyocyte atrophy, apoptosis induction, and decrease in protein synthesis by enhanced autophagy and activation of the ubiquitin-proteasome system lead to cancer-derived myocardial impairment [13].

Despite these advances in our understanding, the multifactorial mechanisms underlying cancer-derived myocardial impairment remain incompletely understood, necessitating further investigations to elucidate the molecular mechanisms and prevent myocardial damage in cancer patients. In this study, we used the mouse cancer cachexia model that we previously established [14] to examine the status of cancer-derived myocardial impairment reported in literature, and validate our model for studying cancer-derived myocardial impairment.

## RESULTS

### A mouse cachexia model using CT26 colon cancer cells

**Figure 1:**
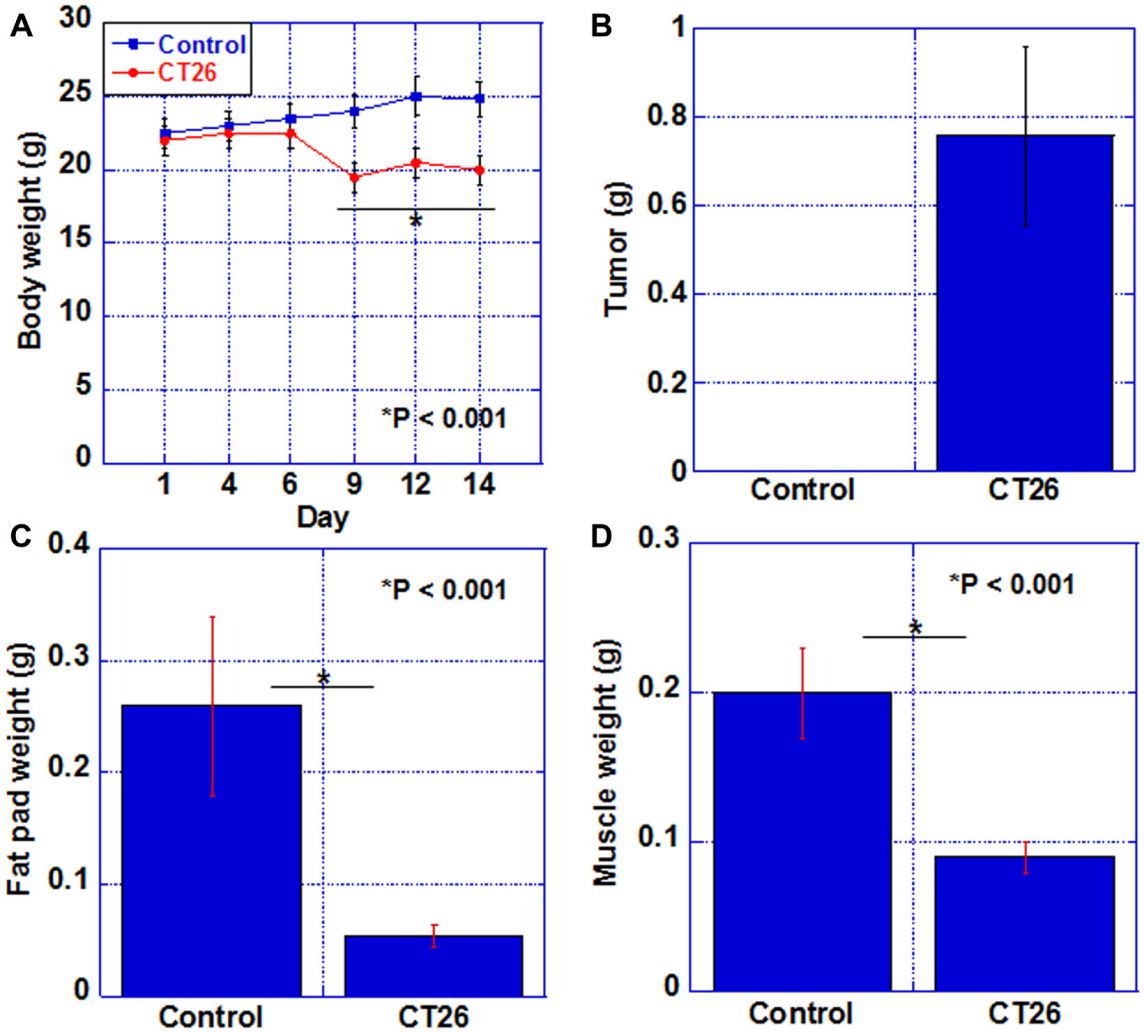
Cachexic features of CT26-inoculated BALB/c mice. (**A**) Body weight, (**B**) tumor weight, (**C**) fat pad weight, and (**D**) weight of QFM (2 muscles per mouse) in each group at euthanasia. Error bar, standard error of data collected from 3 mice. Significant differences were calculated using Student *t*-test. Control, no tumor control; CT26, mice inoculated with CT26 cells intraperitoneally; QFM, quadriceps femoris muscle.

We first established a cachexia model by intraperitoneal inoculation of CT26 mouse colon cancer cells into syngeneic BALB/c mice. As shown in Figure 1, in the cachexia model, the body weight was reduced by approximately 20% compared to that in the control group after day 9. They became moribund on day 14, when euthanasia was performed. Tumor weight in the peritoneum upon euthanasia was 0.78 g (Figure 1B). In the cachexia model, the weight of the fat pad and quadriceps was reduced to 20% and 45%, respectively, as compared to those of the control group (Figure 1C, 1D).

### Alterations in the myocardium in the mouse cachexia model

**Figure 2:**
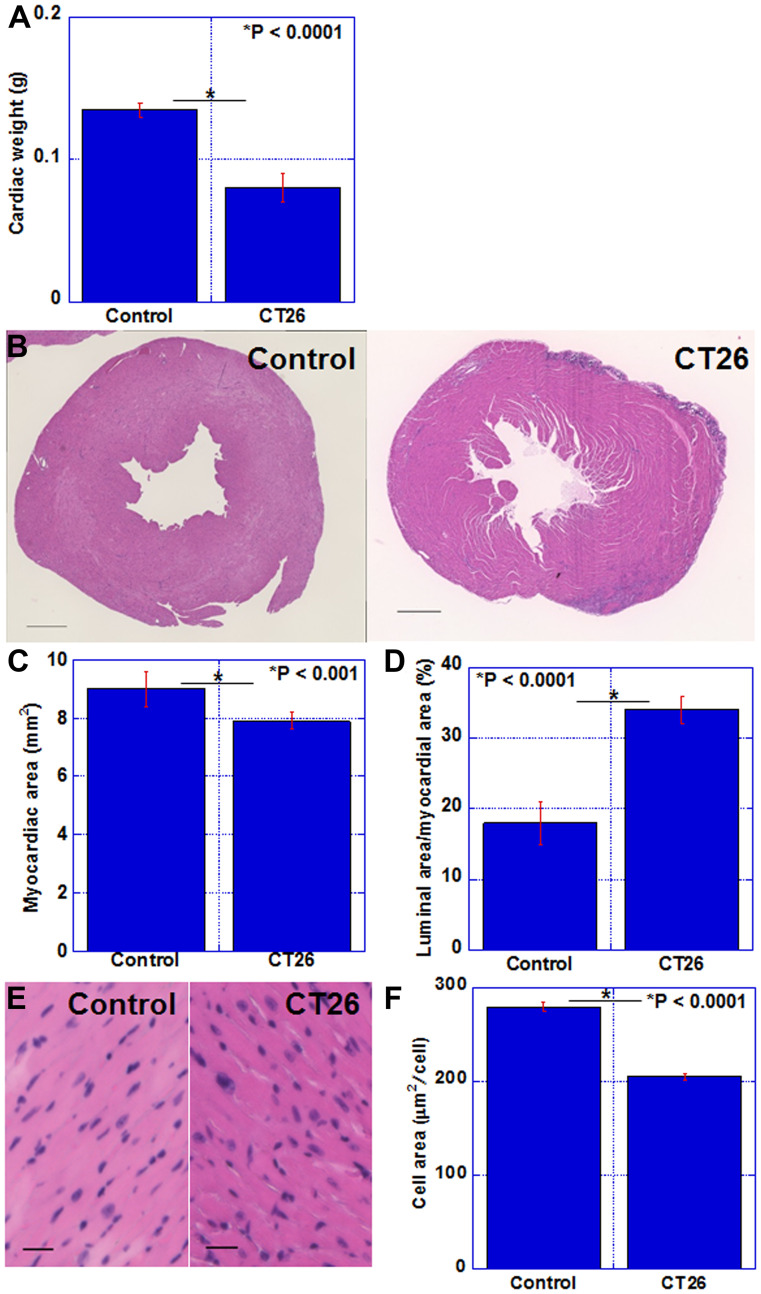
Alterations in the myocardium of CT26-inoculated BALB/c mice. (**A**) Cardiac weight. (**B**) Image of the cut surface of the heart; H&E staining. Scale bar, 0.5 mm (**C**) Myocardial area. (**D**) Percentage of the luminal area to the myocardial area. (**E**) Photomicrogram of the left ventricle. H&E staining. Scale bar, 50 μm. (**F**) Average area of cardiomyocytes. Error bar, standard error of data collected from 3 mice. Significant differences were calculated using Student *t*-test. Control, no tumor control; CT26, mice inoculated with CT26 cells intraperitoneally; H&E, hematoxylin and eosin.

The status of the myocardium in the cachexia model was examined (Figure 2). In the cachexia group, heart weight was reduced to 62% of that in the control group (Figure 2A). A cross section of the heart is shown in Figure 2B. In the cachexia group, the lumen of the left ventricle was dilated. Comparing the cross-sectional myocardial area, the cachexia group showed a reduction to 88% of that in the control group (Figure 2C). The lumen area of the left ventricle increased by 1.9 times (Figure 2D). Figure 2E shows an HE-stained image of the myocardial tissue; notably the nuclear density was increased in the cachexia group. The cardiomyocyte area in the cachexia group was reduced to 72% of that in the control group (Figure 2F). Thus, myocardial atrophy was observed in the cachexia model.

### Alteration in oxidative stress and metabolism-associated proteins

**Figure 3:**
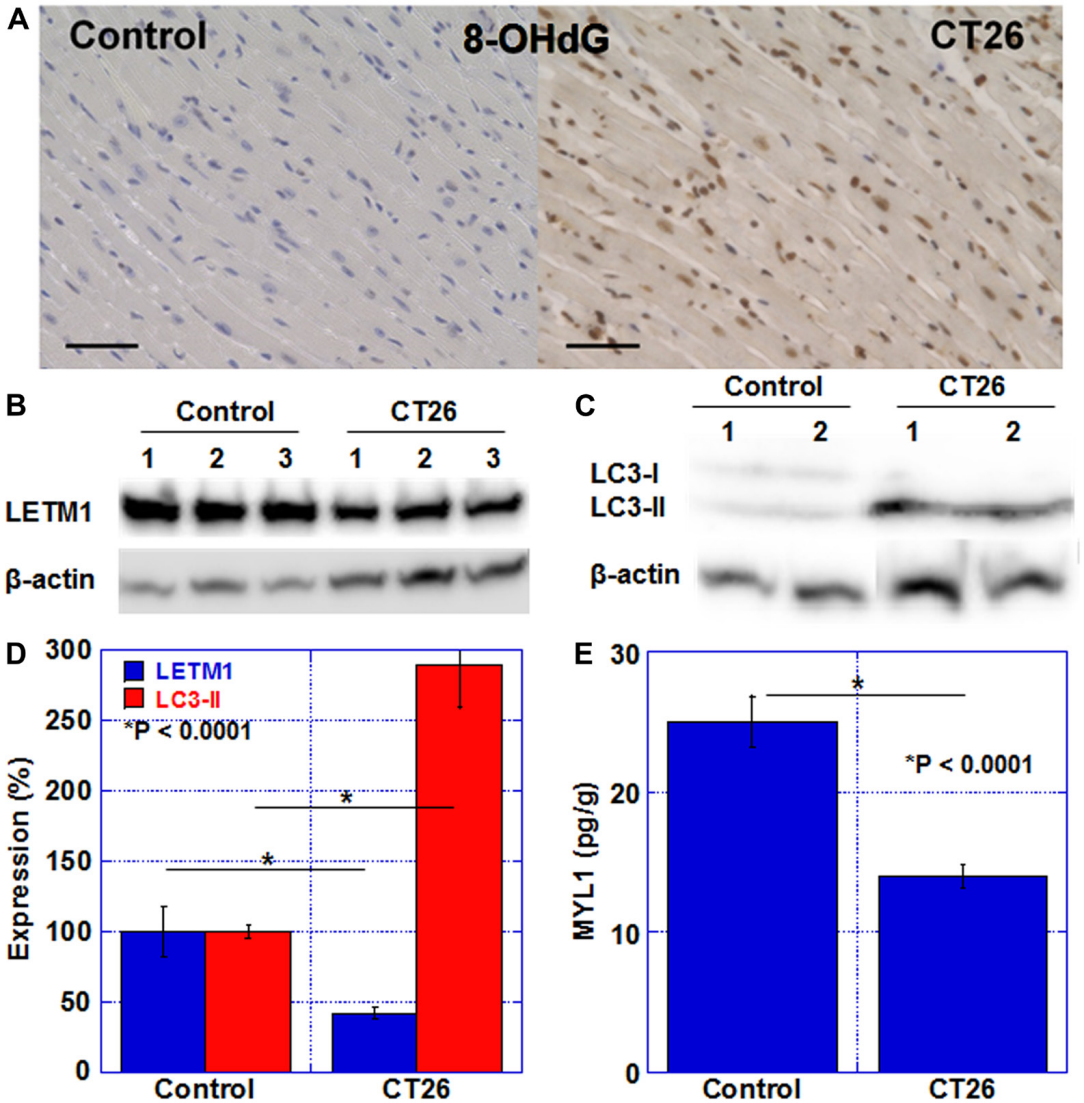
Oxidative stress, mitochondria, and autophagy in the myocardium of CT26-inoculated BALB/c mice. (**A**) Immunostaining of 8-OHdG (oxidative stress). Scale bar, 50 μm. (**B**) Western blot analysis of LETM1 (mitochondria). (**C**) Western blot analysis of LC3. LC3-II is a marker for autophagy. (**D**) Semi-quantification of LETM1 and LC3-II. (**E**) SDS-soluble myosin light chain-2 (MYL1) measured using ELISA. The signals were standardized with the β-actin signal. Error bar, standard error of data collected from 3 mice. Significant differences were calculated using Student *t*-test. 8-OHdG, 8-hydroxy-2′-deoxyguanosine; LETM1, leucine zipper and EF-hand-containing transmembrane protein 1; LC3, microtubule-associated protein light chain-3; Control, no tumor control; CT26, mice inoculated with CT26 cells intraperitoneally; ELISA, enzyme-linked immunosorbent assay.

Next, we examined alterations in oxidative stress and metabolism-related proteins in the myocardium (Figure 3). Oxidative stress in the myocardium was examined with 8-OHdG (Figure 3A). In the control group, the cardiomyocyte nuclei positive rate was 2 ± 0.4%, whereas in the cachexia group, it was 96 ± 1% (*P* < 0.0001). The level of the LETM1 protein, which is located in the mitochondrial inner membrane and involved in crista formation, was examined (Figure 3B and 3D). LETM1 in the myocardium was decreased in all three cachexic mice and was approximately 40% of that in the control group. Furthermore, the protein level of LC3-II, which is an autophagy-related protein, increased by 2.9-fold (Figure 3C, 3E). In addition, SDS-soluble myosin light chain 1 (SDS-MYL1), which indicates maturity of cardiomyocytes, was reduced to 62% in the cachexia group compared to that in the control group. As described above, in cachexia, mitochondrial impairment, autophagy enhancement, and myocardial immaturation were observed in association with increased oxidative stress.

### Alteration in signaling pathways in myocardial cachexia

**Figure 4:**
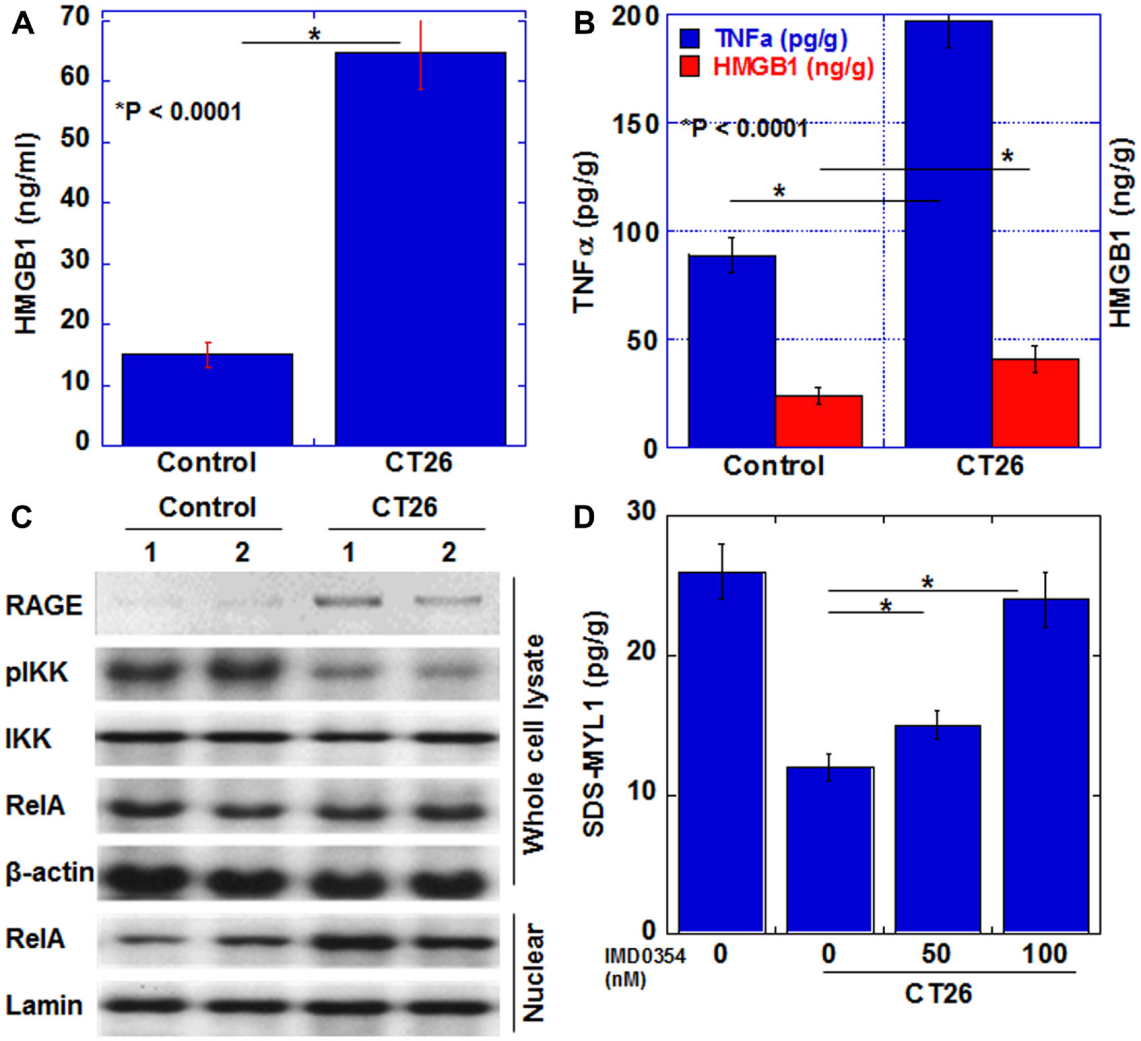
Myocardial cytokines and signals in the CT26-inoculated BALB/c mice. (**A**) Serum HMGB1. (**B**) HMGB1 and TNFα in myocardium. (**C**) Levels of proteins in the signaling pathway of HMGB1 or TNFα. The signals were standardized with the β-actin signal. (**D**) H9c2 cells treated with the ascites of CT26-inoculated BALB/c mice (Ascites) with or without a IKKbeta inhibitor IMD0354. Then SDS-soluble myosin light chain-2 (MYL1) measured using ELISA. Error bar, standard error of data collected from 3 mice. Significant differences were calculated using Student *t*-test. HMGB, high-mobility group box; TNF, tumor necrosis factor; RAGE, receptor for glycation end products; IKK, inhibitor κB kinase; pIKK, phosphorylated IKK; RelA, v-rel avian reticuloendotheliosis viral oncogene homolog A (p65); Control, no tumor control; CT26, mice inoculated with CT26 cells intraperitoneally.

Next, we examined signals associated with myocardial atrophy due to cachexia (Figure 4). In our previous study, we found that serum tumor necrosis factor (TNF)-α and high-mobility group box (HMGB)-1 were increased in cachexia patients [15]. Therefore, we quantified TNFα and HMGB1 in the myocardium (Figure 4A, 4B). In the cachexia group, HMGB1 and TNFα increased by 1.8 times and 2.3 times, respectively, compared to those in the control group. Next, the expression of signal-related proteins was examined (Figure 4C). Expression of RAGE, a receptor for HMGB1, was increased in the cachexia group. Moreover, when NFkB, which is a signal transduction system common to TNFα and HMGB1, was examined, the phosphorylation level of IKK was decreased and the nuclear RelA level was increased. These findings indicated the activation of the NFkB signal in the cachexia group. To confirm the significance of NFkB signal in cancer-derived myocardial disorder, we examined maturity of H9c2 cells by measuring SDS-MYL1 after H9c2 cells exposed to CT26-inoculated mouse ascites with or without an IKKbeta inhibitor, IMD0354 (Figure 4D). SDS-MYL1 was decreased by ascites; however, IMD0354 treatment recovered SDS-MYL1 levels in a dose-dependent manner.

### Alteration in energy metabolism in myocardial cachexia

**Figure 5:**
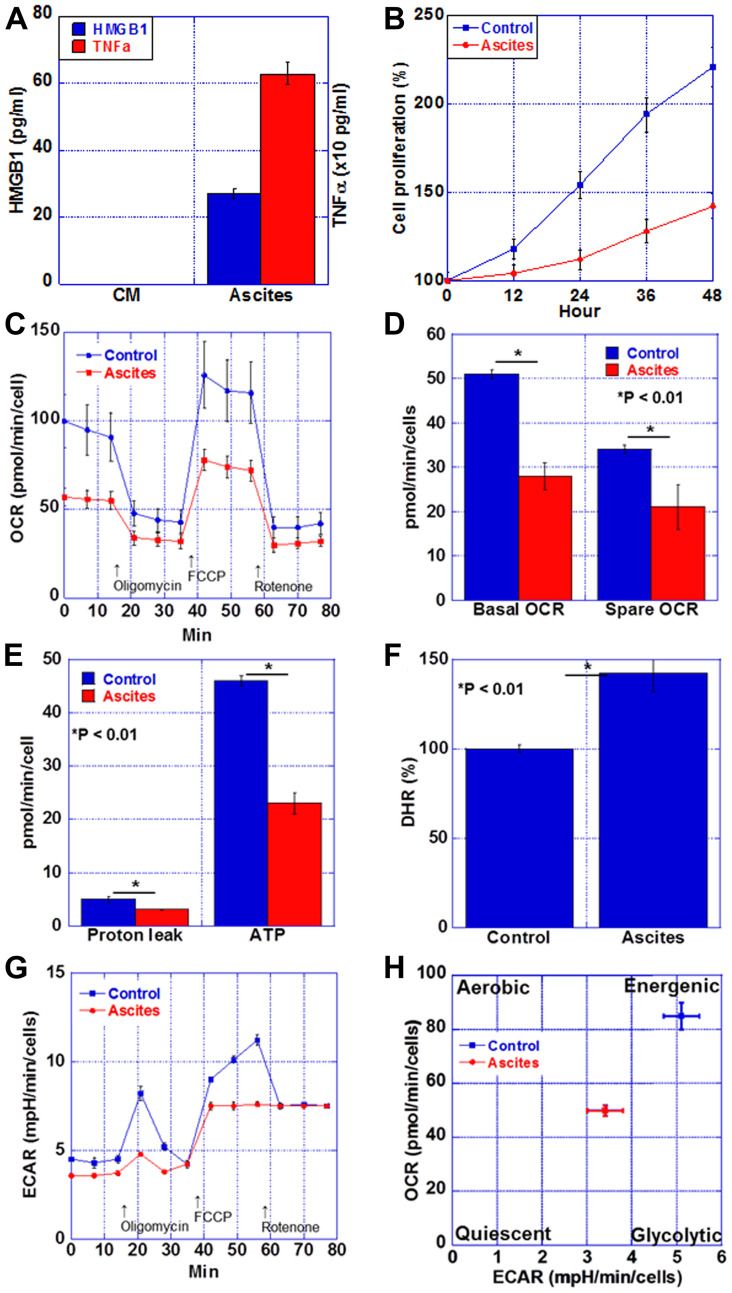
Energy metabolism in the myocardium of CT26-inoculated BALB/c mice. (**A**) Protein levels of HMGB1 and TNFα in the ascites of CT26-inoculated BALB/c mice or H9c2 cultured medium (CM). (**B**) Cell proliferation of H9c2 cells treated with the ascites of CT26-inoculated BALB/c mice (Ascites) or H9c2 cultured medium (Control). (**C**) Mitochondrial stress test (Seahorse assay) of H9c2 cells. (**D**) Basal OCR and spare OCR. (**E**) Proton leak and ATP production. (**F**) Oxidative stress examined by DHR. (**G**) Glycolytic stress test of H9c2 cells. (**H**) Matrix analysis of OCR and ECAR. Error bar, standard error from 3 mice. Significant differences were calculated using Student *t*-test. HMGB, high-mobility group box; TNF, tumor necrosis factor; OCR, oxygen consumption rate; ECAR, extracellular acidification rate; DHR, dihydrorhodamine 123.

Finally, we examined energy metabolism in cardiomyocytes in the cachexia group (Figure 5). We stimulated myocardial cachexia by treating rat cardiomyocytes, H9c2, with the mouse cachexia model ascites fluid. The levels of TNFα and HMGB1 in the culture medium used for treatment in each of the control group and the ascites treatment group were examined. As shown in Figure 5A, TNFα and HMGB1 were below the detection limit in the control group, whereas the levels of both in the ascites-treated group were high. In addition, cardiomyocyte proliferation was suppressed in the ascites-treated group (Figure 5B). Next, the respiratory status of cardiomyocytes was examined by flux analysis (Figure 5C–5E). Respiratory quotients in the ascites-treated cells were lower at basal and spare levels than those in the controls. Furthermore, proton leak and ATP level were lower in the ascites-treated cells than in the control cells. When the reactive oxygen species levels in the myocardium were examined with dihydrorhodamine 123, it was found to be increased by 1.4 times in the ascites-treated cells (Figure 5F). Thus, oxidative phosphorylation was decreased in the ascites-treated cardiomyocytes. In contrast, glycolysis was decreased in the ascites-treated cells compared to that in the controls (Figure 5G). Matrix analysis revealed that a quiescent state was induced in the ascites-treated cardiomyocytes (Figure 5H).

## DISCUSSION

In this study, the cachexia mouse model revealed morphologically decreased heart weight, decreased volume of the myocardium, and dilated LV cavity. These findings correspond to those of LV failure in humans. Similar findings have been found in previous reports. In the rodent cachexia model, thinning of the LV septum, LV dilation, impaired systole, and myocardial fibrosis are observed [16, 17]. In our model, however, myocardial fibrosis was not found. To form fibrosis, longer time course should be needed. In addition, progressive decrease in LV myocardial mass, decrease in pump function, elevation in cardiovascular neurohormone, and increase in protein degeneration are observed [18, 19]. Functionally, reduced contraction and relaxation are also seen at the cardiomyocyte level [19].

In a rat cachexia model, it has been reported that DNA damage induced by reactive oxygen species causes widespread systemic organ damage [20]. In this study, we examined levels of 8-OHdG, a by-product of DNA injury due to oxidative stress, and found that cachexia mice frequently accumulated 8-OHdG in the nucleus of the cardiomyocytes. 8-OHdG has attracted attention as a marker of heart failure, and it has been revealed that it increases with a higher class of the New York Heart Association Classification [21, 22]. Oxidative stress that produces 8-OHdG occurs in cachexia due to various reasons such as chemotherapy, consumption of antioxidants, and chronic inflammation, leading to heart failure [10]. Furthermore, oxidative stress is observed in tumor-bearing rat models as well as oxidation of proteins involved in glycolysis, ATP production, muscle contraction, and mitochondrial function [23]. In skeletal muscle, oxidative stress has been reported to be involved in the atrophy of type 2 muscle fibers [23], and oxidative stress is also important as a cause of atrophy in the myocardium.

There are few reports on alterations in myocardial mitochondria in cachexia. Our results showed that mitochondria were reduced, and both oxidative phosphorylation and glycolysis were suppressed. As a result, myocardial energy metabolism in cachexia became quiescent. Similar to our results, Sjöström et al. reported a decrease in mitochondria in cardiomyocytes in a murine cachexia model [24]. In skeletal muscle atrophy, mitochondrial dysfunction occurs at an early stage. Furthermore, there is a decrease in ATP production, mitochondrial biogenesis, and mitochondrial quality control, along with enhanced mitophagy and apoptosis [25]. From these findings, it is expected that similar changes will occur in the myocardium. In addition, myocardial toxicity by ethanol causes increased oxidative stress and induction of autophagy [26]. In our experiments, increased oxidative stress and autophagy were observed, consistent with these reports. Although no increase in apoptosis was confirmed in our model (data not shown), persistent cachexia might cause apoptosis in cardiomyocytes.

We examined the amount of LETM1, a structural protein of mitochondria, in the myocardium of the cachexia model and found that it was decreased. As autophagy is enhanced, mitophagy-induced mitochondrial loss might be the cause. LETM1 is located in the inner mitochondrial membrane and its deficiency is known to lead to Wolf-Hirschhorn syndrome [27]. LETM1 is thought to be involved in the mitochondrial network, metabolic function, and regulation of cell death [28]. Cells with LETM1 knockdown show inhibition in mitochondrial calcium transport and decrease in Mn-superoxide dismutase expression and aconitase activity [27]. Oxidative phosphorylation is also suppressed by disorders of complexes 2 and 4 of the electron transport chain [27]. The decrease in the LETM1 protein level observed in our cachexic mice model might cause similar mitochondrial disorders.

In myocardial remodeling with cancer cachexia, altered gene expression is reported. For example, it is known that BNP and c-Fos increase, peroxisome proliferator-activated receptorα and carnitine palmitoyltransferase-1β decrease, and major histocompatibility complex (MHC) and glucose transporter (GLUT) isoforms change from the adult type (MHCα, GLUT4) to the fetal type (MHCβ and GLUT1) [17]. In addition, expression of matrix metalloproteinase (MMP)-2, MMP-3, MMP-9, and MMP-14 is enhanced, and subsequently, collagen turnover is promoted. From these findings, it is considered that myocardial fibrosis is induced [29]. In myocardial cells, there is a decrease in myosin and myofibrils, which corresponds to decreased cardiac function [24].

In our mouse cachexia model, TNFα and HMGB1 in ascites and serum were increased [14]. These proteins are also found to increase in the blood of cachexia patients with colorectal cancer [15]. Furthermore, our data confirmed the activation of NFkB, a common signaling pathway associated with both proteins in the myocardium. NFkB promotes the expression of anti-apoptotic genes and suppresses apoptosis in skeletal muscle [30]. Furthermore, TNFα also suppresses the expression of Mn-SOD and catalase in cardiomyocytes and decreases glutathione peroxidase [31], leading to increased intracellular active oxygen and lipid peroxide and consequent cell damage [31]. Furthermore, we treated rat cardiomyoblasts with ascitic fluid from the mouse cachexia model. In our experiments, species-specific phenotype between mouse and rat was observed in case of TNFα, but it is considered that HMGB1 is not affected by species differences. As HMGB1 induces the expression of inflammatory cytokines such as TNFα [32, 33], it is considered that HMGB1 in mouse ascites induced rat TNFα expression in rat cardiomyoblasts. As a result, it was considered that oxidative stress increased.

In summary, our established mouse cachexia model showed various myocardial changes associated with cancer cachexia such as oxidative stress in the myocardium, energy metabolism, autophagy, and inflammatory cytokines. Therefore, we propose the use of this model for future investigations of cancerous myocardial damage. We have revealed that combined feeding with laurate and glucose improves cancer sarcopenia [14]. Using this cancer-derived myocardial impairment model, we attempt to assess the effect of the diet supplemented with laurate and glucose.

## MATERIALS AND METHODS

### Cell culture

The CT26 mouse colon cancer cell line was obtained as a kind gift from Professor I. J. Fidler (MD Anderson Cancer Center, Houston, TX, USA). CT26 cells were cultured in Dulbecco’s modified Eagle’s medium (DMEM; Wako Pure Chemical Industries, Ltd., Osaka, Japan) supplemented with 10% fetal bovine serum (Sigma-Aldrich Chemical Co., St. Louis, MO, USA). Embryonic rat heart-derived H9c2 myocardial cells were purchased from American Type Culture Collection (Manassas, VA, USA). H9c2 cells were cultured in high-glucose DMEM with 10% fetal bovine serum (Sigma-Aldrich). An IKKbeta inhibitor, IMD0354 (ab144823, Abcam, Cambridge, MA, USA) was purchased.

### Animals

Five-week-old male BALB/c mice were purchased from SLC Japan (Shizuoka, Japan). The animals were maintained in a pathogen-free animal facility under a 12/12-h light/dark cycle at a temperature (22°C)- and humidity-controlled environment, in accordance with the institutional guidelines approved by the Committee for Animal Experimentation of Nara Medical University, Kashihara, Japan, following current regulations and standards of the Japanese Ministry of Health, Labor and Welfare (approval nos. 11812, 11857, 11916, 12043 and 12262). Animals were acclimated to their housing for seven days before the start of the experiment. Mice were fed with a CE-2 diet (containing 5% crude fat, mainly derived from soy bean oil; CLEA Japan, Inc., Tokyo, Japan).

To measure tumor weight, mice were euthanized by aortic blood removal under anesthesia sevoflurane (Maruishi Pharmaceutical Co. Ltd., Osaka, Japan) with and the peritoneal tumors were dissected from the intestine, mesenterium, diaphragm, and abdominal wall, grossly removing non-tumoral tissues. For preparation of the heart, after euthanasia, the heart was excised and its weight was measured, then divided into two at 2/5 from the apex of the heart. The upper part was used for histological analysis, and the lower part was used for analyzing expressed proteins.

For preparation of skeletal muscles, the quadriceps femoris muscle (QFM) was cut at the muscle end on the upper edge of the patella, peeled off from the femur, and separated at the muscle origin on the frontal surface of the anterior lower iliac spine. The excised QFM was weighed immediately, avoiding drying. After measurement, QFM was stored at -80°C.

### Histological analysis

Myocardial tissues were fixed in 4% paraformaldehyde, dehydrated, and embedded in paraffin. After slicing the created block to 3 μm, we performed hematoxylin and eosin (H&E) and immunohistochemical staining to observe the morphology, accumulation of oxidative stress, and expression of mitochondria in the myocardial tissue. Anti-8-hydroxy-2′-deoxyguanosine (8-OHdG) antibody (Japan Institute for the Control of Aging, NIKKEN SEIL Co., Ltd., Shizuoka, Japan) was used to confirm the accumulation of oxidative stress in the nuclei. The antibody was used at a concentration of 1.0 μg/mL. Secondary antibodies (Medical and Biological Laboratories, Nagoya, Japan) were used at a concentration of 0.2 μg/mL. Tissue sections were color-developed with diamine benzidine hydrochloride (Dako, Glostrup, Denmark) and counterstained with Meyer’s hematoxylin (Sigma-Aldrich Chemical Co., St. Louis, MO, United States) for visualization of the nuclei. Regarding accumulation of oxidative stress, the number of positive cells was counted. These histological analyses were verified using a fluorescence microscope (BZ-X710, Keyence, Osaka, Japan).

### Protein extraction

The lower part of the excised heart stored at –80°C was crushed with a hammer to remove tendons and fascia. Only the muscle tissue was washed with cold phosphate-buffered saline and pelleted with a sonicator (QSONICA, WakenBtech Co. Ltd., Kyoto, Japan). Whole-cell lysates were prepared using 0.1% SDS-added RIPA-buffer as previously described (Thermo Fisher Scientific, Tokyo, Japan) [34]. Protein assay was performed using a Protein Assay Rapid Kit (Wako Pure Chemical Corporation, Osaka, Japan).

### Immunoblot analysis

Lysates were separated using 10% sodium dodecyl sulfate (SDS)-polyacrylamide gel electrophoresis and transferred onto nitrocellulose membranes. The membranes were then incubated with primary antibodies specific to LETM1 (16024-1-AP; Proteintech Group, Inc., Rosemont, IL, USA), LC3 (CTB-LC3-1-50; Cosmo Bio Co. Ltd., Tokyo, Japan), RAGE (SC-365154, A-9; Santa-Cruz Biotechnology, Santa-Cruz, CA, USA), IkB kinase (IKK; 15649-1-AP; Proteintech), phosphorylated IKKα/β (A044, pSer180/181; Assay Biotech, Fremont, CA, USA), and RelA (13629-1-AP; Proteintech), followed by peroxidase-conjugated IgG antibodies (P0217; Dako). Anti-β-actin antibody (sc47778, C0817; Santa-Cruz) and anti-lamin B1 antibody (66095-1-IG; Proteintech) were used for loading controls. Immune complexes were visualized using the Fusion Solo imaging system (M&S Instruments Inc., Osaka, Japan).

### Enzyme-linked immunosorbent assay (ELISA)

ELISA kits were used to measure the concentration of MYL1 (Cusabio Biotech Co., Ltd., Houston, United States), HMGB1 (Shino-Test Co., Sagamihara, Japan), and human tumor necrosis factor (TNF)-α, according to the manufacturers' instructions. For measurement, whole-cell lysate was used.

### Mitochondrial stress test (Seahorse assay)

To simulate the cachectic condition, the ascites of CT26-induced cachexia mice were collected. The ascites were filtered with the Sterile Millex Filter (pore size 0.22 μm, Sigma). H9c2 cells were cultured in a regular medium for 48 h and this culture medium was also filtered. The H9c2 cells were cultured in a growth medium in 6-well plates before the Seahorse assay with the ascites (20% v/v) or the collected cultured medium (20% v/v). Oxygen consumption rates (OCR) of 1 × 10^4^ viable H9c2 cells per well were measured using the Seahorse XFe24 Extracellular Flux Analyzer with Seahorse XF24 FluxPaks (Agilent Technologies, Chicopee, Canada). Seahorse assays were carried out as follows: OCR in pmol/min were measured (basal OCR) before and after successive injection of 1-μM oligomycin (ATP synthase inhibitor), 2-μM FCCP (carbonyl cyanide-p-trifluoromethoxy phenylhydrazone, an uncoupling protonophore), 1-μM rotenone (Complex I inhibitor), and 5-μM antimycin A (Complex III inhibitor). From the resulting data, we determined the OCR associated with respiratory ATP synthesis (oligomycin-sensitive), the maximum OCR in FCCP-uncoupled mitochondria and the rotenone-sensitive OCR attributable to uncoupled Complex I activity, the antimycin-sensitive Complex II/III activity, the OCR by mitochondrial functions other than ATP synthesis that are mitochondrial membrane potential-driven (proton leak), non-respiratory oxygen consumption, and the respiratory “spare capacity” (excess capacity of the respiratory electron transport chain that is not being used in basal respiration).

### Glycolytic stress test

The extracellular acidification rate (ECAR) of H9c2 cells was measured using a modified glycolytic stress test in the Seahorse XFe24 Extracellular Flux Analyzer with Seahorse XF24 FluxPaks (Agilent Technologies, Chicopee, Canada). H9c2 cells were cultured in a growth medium in 6-well plates with the ascites or the cultured medium before Seahorse experiments. H9c2 cells (1 × 10^4^ cells/well) were later plated in the XF base medium (Agilent Technologies, Chicopee, Canada) containing 200 mM L-glutamine and 5 mM HEPES, as recommended by the manufacturer for glycolytic assays. The sensor cartridge apparatus was rehydrated one day in advance by adding 1-mL XF Calibrant to each well and incubating at 37°C until needed. The injection ports of the sensor cartridge apparatus were loaded with the following drugs, in chronological order of four injections, to meet the indicated final concentrations in the wells: 10 mM glucose, 1 μM oligomycin, 1 μM rotenone and 5 μM antimycin A (combined injection), and 50 mM 2-deoxyglucose. Treatment with the rotenone/antimycin combination allowed assessment of the impact of electron transport on ECAR by respiratory acidification coupled to passage of some glycolytic pyruvate through the TCA cycle to supply respiration.

### Statistical analysis

Statistical significance was calculated using unpaired Student *t*-tests using InStat software (version 3.0; GraphPad Software, Inc., La Jolla, CA, United States). Data are expressed as the mean ± standard deviation of three independent experiments. *P* < 0.05 (two-sided) was considered to indicate statistical significance.
